# Suppression of lung inflammation by the ethanol extract of Chung-Sang and the possible role of Nrf2

**DOI:** 10.1186/s12906-018-2422-3

**Published:** 2019-01-10

**Authors:** Jin-Woo Han, Kyun Ha Kim, Min-Jung Kwun, Jun-Yong Choi, Sang-Jun Kim, Seung-Il Jeong, Beom-Joon Lee, Kwan-Il Kim, Ran Won, Ji Hoon Jung, Hee Jae Jung, Myungsoo Joo

**Affiliations:** 1 Division of Allergy, Immune and Respiratory System, Department of Internal Medicine, College of Korean Medicine, Kyung Hee University, Kyungheedae-ro 23, Seoul, 02447 Republic of Korea; 2 Division of Applied Medicine, School of Korean Medicine, Pusan National University, PNU Rd 49, Yangsan, 50612 Republic of Korea; 3 Department of Internal Medicine, Korean Medicine Hospital, Pusan National University, Yangsan, 50612 Republic of Korea; 4 Jeonju AgroBio-Materials Institute, Jeonju, 57810 Republic of Korea; 5 Department of Clinical Korean Medicine, College of Korean Medicine, Kyung Hee University, Seoul, 130-701 Republic of Korea; 6 Division of Health Sciences, Department of Biomedical Laboratory Science, Dongseo University, Busan, 47011 Republic of Korea; 7 Department of Biochemistry and Molecular Biology, Tulane University School of Medicine, New Orleans, LA 70112 USA

**Keywords:** Formulation of herbal remedies, Asian traditional medicine, Lung inflammation, Nrf2, NF-κB

## Abstract

**Background:**

Asian traditional herbal remedies are typically a concoction of a major and several complementary herbs. While balancing out any adverse effect of the major herb, the complementary herbs could dilute the efficacy of the major herb, resulting in a suboptimal therapeutic effect of an herbal remedy. Here, we formulated Chung-Sang (CS) by collating five major herbs, which are used against inflammatory diseases, and tested whether an experimental formula composed of only major herbs is effective in suppressing inflammation without significant side effects.

**Methods:**

The 50% ethanol extract of CS (eCS) was fingerprinted by HPLC. Cytotoxicity to RAW 264.7 cells was determined by an MTT assay and a flow cytometer. Nuclear NF-κB and Nrf2 were analyzed by western blot. Ubiquitinated Nrf2 was similarly analyzed following immunoprecipitation of Nrf2. Acute lung inflammation and sepsis were induced in C57BL/6 mice. The effects of eCS on lung disease were measured by HE staining of lung sections, a differential cell counting of bronchoalveolar lavage fluid, a myeloperoxidase (MPO) assay, a real-time qPCR, and Kaplan-Meier survival of mice.

**Results:**

eCS neither elicited cytotoxicity nor reactive oxygen species. While not suppressing NF-κB, eCS activated Nrf2, reduced the ubiquitination of Nrf2, and consequently induced the expression of Nrf2-dependent genes. In an acute lung inflammation mouse model, an intratracheal (i.t.) eCS suppressed neutrophil infiltration, the expression of inflammatory cytokine genes, and MPO activity. In a sepsis mouse model, a single i.t. eCS was sufficient to significantly decrease mouse mortality.

**Conclusions:**

eCS could suppress severe lung inflammation in mice. This effect seemed to associate with eCS activating Nrf2. Our findings suggest that herbal remedies consisting of only major herbs are worth considering.

## Background

Cold, allergic rhinitis, pneumonia, and asthma are common respiratory diseases rampant in human, which casually accompany pulmonary inflammation. The pulmonary inflammation can be caused by commensal infections by bacteria. For example, lipopolysaccharide (LPS), a cell-wall component of *Escherichia coli,* functions as a pathogen-associated molecular pattern (PAMP) molecule, triggering an inflammatory response [1]. LPS binding to TLR4 activates a signaling, resulting in activation of NF-κB [2]. Activated NF-κB is largely responsible for the production of cytokines including tumor necrosis factor-α (TNF-α), interleukin (IL)-1β, and IL-6 [3]. These cytokines play a key role in propagating the inflammatory reaction, including the recruitment of neutrophils to the lungs [4]. Since suppressing inflammatory responses often results in positive outcomes, therapeutics against NF-κB activity have been developed [5].

While inflammation is critical in innate immunity, inordinate inflammatory reaction inflicts damages to host organs [6]. For instance, TLR4 signaling triggered by LPS induces the production of intracellular reactive oxygen species (ROS) [7], which could damage lung parenchyma, exacerbating lung inflammation [8]. In this oxidative environment, ROS inactivate Keap1 [9]. As Keap1 mediates the constitutive ubiquitination, and thus the constant degradation, of Nrf2, ROS blocking Keap1 decreases the ubiquitination of Nrf2, resulting in the accumulation of Nrf2 in the nucleus, indicative of Nrf2 activation [10]. Nuclear Nrf2 induces the expression of NQO-1 (NAD(P)H:quinone oxidoreductase 1), GCLC (glutamate-cysteine ligase catalytic subunit), and HO-1 (heme oxygenase-1) [11]. It is well-documented that activated Nrf2 protects mice from various inflammatory pulmonary diseases, such as acute pulmonary injury, smoke-induced emphysema, and asthma [12–14]. Therefore, along with NF-κB, Nrf2 has gained an attention as a potential therapeutic target for diseases closely linked with inflammation [14, 15].

Herbal remedies have been a pillar in practicing Asian traditional medicine, including Korean traditional medicine (KTM). Typically, an herbal remedy is composed of a key herb, which executes a major pharmacologic effect at the target symptom, and of secondary, complementary herbs, which play a role in subduing an adverse effect of the major herb [16]. However, it is possible that while the complementary herbs contribute to reducing an adverse effect of the major herb, the pharmaceutical efficacy of the major herb could be diluted out by accompanying complementary herbs, resulting in a suboptimal efficacy of the major herb. This possibility prompted us to test whether an herbal formula composed of major herbs only can be effective without showing a significant side effect.

To test this possibility, we formulated an experimental herbal remedy, named Chung-Sang (CS), which comprises five major herbs. One of the constituents of CS, *Caryophyllus aromaticus* L., has shown to have an anti-bacterial effect [17, 18]. Other four constituents, *Mentha haplocalyx* Briq. [19], *Magnolia biondii* Pamp. [18], *Xanthium sibiricum* Patr. [18], and *Asarum sieboldii* Miq. [18] have been prescribed to relieve inflammatory respiratory symptoms. With the 50% ethanol extract of CS (eCS), we tested whether eCS suppresses lung inflammation without significant side effects. Here, we show that eCS suppressed neutrophilic lung inflammation in mice and that a single administration of eCS effectively decreased the septic shock of mice. Mechanistic experiments suggest that these effects were associated with Nrf2 activated by eCS. Our findings could provide evidence that a new formula composed of only major herbs can be developed as an alternative to the traditional herbal remedy.

## Methods

### Ethanol extraction of Chung-Sang (eCS)

The herbs comprising Chung-Sang (CS) (Table 1) were procured from Kwang- Myoung-Dang (Pusan, Republic of Korea). CS is stored in the Korean Medicinal herbarium at Pusan National University. The amount of each herb of CS was based on a daily dose typically prescribed to patients. For the ethanol extract of CS (eCS), 500 g of CS was mixed with 5 L of 50% ethanol at 58 °C overnight, which yielded 6 g of powder. Phosphate buffered saline (PBS) buffer was added to the power, which was through a 0.2 μm filter.

**Table 1:** Composition of Chung-Sang

Scientific name	Herbal name	Amount (g)
*Mentha haplocalyx* Briq.	Menthae haplocalycis Herba(薄荷)	10
*Magnolia biondii* Pamp	Magnoliae Flosis(辛夷)	5
*Xanthium sibiricum* Patr*.*	Xanthii Fructus(蒼耳子)	5
*Asarum sieboldii* Miq.	Herba Asari(細辛)	5
*Caryophyllus aromaticus* L.	Caryphylli Flos(丁香)	5
Total		30

### Fingerprinting analysis of eCS

Fingerprinting eCS was performed as described elsewhere [20], with the mobile phase composed of 0.1% formic acid (A) and acetonitrile (B) in water. The conditions of solvent gradient elution were 20% B in 0–3 min, 20% B in 5 min, 30% B in 12 min, 35% B in 16 min, 60% B in 20 min, 80% B in 30 min, 80% B in 34 min, 60% B in 37 min, 20% B in 40 min. Fifteen μL of eCS was run at the flow rate of 0.5 mL/min and 37 °C. All the chemicals were detected at wavelengths of 254 ~ 360 nm. The retention time of each chemical was compared to those of standard chemicals for identification. Chemical standards, such as chlorogenic acid, rosmarinic acid, Eugenol, 6-Gingerol, and aristolochic acid I, were obtained from Sigma-Aldrich (Seoul, Korea).

### Reagents and antibodies

LPS (*E. coli* O55:B5, Alexis Biochemical, CA, USA), MG132 (Merck Millipore, MA, USA), and sulforaphane and d-(+)-galactosamine hydrochloride (Sigma-Aldrich) were used for the study. Except for anti-V5 (Thermo Fisher Scientific, Seoul, Korea) and ant-HA and anti-Flag antibodies (Sigma-Aldrich), all the antibodies were procured from Santa Cruz Biotechnology, CA, USA.

### Cell culture

The culture condition of RAW 264.7 (ATCC, MD, USA) was described elsewhere [20]. Specified otherwise, cells were cultured in a standard CO_2_ humidified incubator.

### Measurement of cytotoxicity

Possible toxicity on the cell, which could be elicited by eCS, was determined by an MTT assay (vybrant MTT assay kit, Thermo Fisher Scientific). Live cells were calculated as described previously [20]. Each experiment was set in triplicate and performed three times independently.

### Measurement of intracellular reactive oxygen species (ROS)

As described in a previous study [20], RAW 264.7 cells (1 × 10^6^ cells/well) were incubated with carboxy-H_2_DCFDA (Molecular Probes, Eugene, OR, USA; 100 μM, 30 min, 37 °C). Data were acquired and analyzed by the BD FACS Canto II system (BD Biosciences, CA, USA) and FlowJo (Tree Star, San Carlos, CA, USA), respectively.

### Western blot analysis

Total and nuclear proteins were isolated by 0.5% NP-40 lysis buffer and NE-PER nuclear extraction kit, respectively, as instructed by the protocol of the manufacturer (Thermo Fisher Scientific). After being quantitated by Bradford (Bio-Rad), 50 μg of proteins were run on 7 to 8% NuPAGE gel in MOPS running buffer (Thermo Fisher Scientific). Proteins on the gel were transferred to PVDF membrane by a semi-dry blotter (Bio-Rad). The membrane was incubated with antibodies for 1 h at room temperature. The band of interest was revealed after being incubated with HRP-conjugated secondary antibodies for 1 h at room temperature and chemiluminescence (SuperSignal® West Femto, Thermo Scientific).

### Ubiquitination analysis

HEK 293 cells transfected with plasmids that encode HA-Ub, V5-Nrf2, and Flag-Keap1, were treated with eCS (0.1 μg, 16 h), with or without MG132 (5 μM, 2 h). Nrf2 was precipitated with 1 μg of the anti-V5 antibody, the complex of which was pulled down with protein A-sepharose (Thermo Fisher Scientific) and immunoblotted with the anti-HA antibody for revealing the ubiquitinated Nrf2.

### Isolation of total RNA, semi-quantitative RT-PCR, and real-time quantitative PCR

QIAGEN RNeasy®mini kit and the protocol of the manufacturer (Qiagen, Germany) were employed to extract total RNA from cells or lung tissue. Two μg of RNA was reverse-transcribed to cDNA (Fisher Scientific), which was subject to an end-point dilution including three serial dilutions (1:1, 1:5, 1:25, and 1:125). cDNA was amplified with TaKaRa PCR kit (Takara Bio, Shiga, Japan) and a series of the forward and reverse primers. NQO-1 was amplified with 5’-GCAGTGCTTTCCATCACCC-3′ and 5’-TGGAGTGTGCCCAATGCTAT-3′; HO-1 was with 5’-TGAAGGAGGCCACCAAGGAGG-3′ and 5’-AGAGGTCACCCAGG TAGCGGG-3′; GCLC was with 5’-CACTGCCAGAACACAGACCC-3′ and 5’-ATGGTCTG GCTGAGAAGCCT-3′; and GAPDH was with 5’-GGAGCCAAAAGGGTCATCAT-3′ and 5’-GTGATGGCATGGACTGTGGT-3′. PCR started at 95 °C for 5 min, followed by 25 cycles of denaturation (95 °C, 30 s), annealing (55 °C, 30 s), and extension (72 °C, 40 s), along with a single extension (72 °C, 7 min). DNA synthesized by PCR was run on 1.5% agarose gels in TBE buffer (100 V, 30 min), which was stained with GRgreen (Biolabo, châtel-St-Denis, Switzerland) and visualized by an LED light. Compared to an internal control, glyceraldehyde-3-phosphate dehydrogenase (GAPDH), the expressed genes were quantitated by Image J software (NIH; Bethesda, MD, USA).

Similarly, 1 μg of total RNA was reverse-transcribed for a real-time qPCR. PCR was performed with SYBR Green PCR Master Mix (Enzynomics, Daejeon, Korea). TNF-α was analyzed by 5′-GGTCTGGGCCATAGAACTGA-3′ and 5′-CAGCCTCTTCTCATTCCTGC-3′; IL-1β was by 5′- AGGTCAAAGGTTTGGAAGCA-3′ and 5′-TGAAGCAGCTATGGCAA CTG-3′; IL-6 was by 5′- TGGTACTCCAGAAGACCAGAGG-3′ and 5′- AACGATGATGCA CTTGCAGA-3′; and GAPDH was by 5′-TTGATGGCAACAATCTCCAC-3′ and 5′-CGTCCC GTAGACAAAATGGT-3′. PCR started at 95 °C for 10 min, followed by 40 cycles of 95 °C for 10 s, 57 °C for 15 s, and 72 °C for the 20 s. The reaction was carried out in a Rotor-Gene Q real-time PCR system (Qiagen). The threshold cycles (Ct) were used to quantify the target genes.

### Acute neutrophil inflammation mouse model and survival study

C57BL/6 mice (Samtaco Bio Korea, Korea) were used to induce neutrophilic lung inflammation. The detailed procedure was described elsewhere (). In brief, mice (*n* = 5/group) were injected with a single intratracheal (i.t.) LPS and 2 h later with a single i.t. eCS (0.1 mg/kg or 1 mg/kg body weight (b.w.)). At 24 h after LPS treatment, bilateral bronchoalveolar lavage (BAL) was performed to obtain BAL fluid (BALF). Cells in BALF were harvested and stained with Hemacolor (Merck, Darmstadt, Germany). One hundred cells per microscopic field were counted and 300 cells in total were analyzed. After perfusion, mouse lungs were fixed, embedded in paraffin, and stained with hematoxylin and eosin (HE). From a mouse, three discrete lung sections were examined in 200X microscopic magnifications.

For obtaining Kaplan-Meier survival ratio, mice (*n* = 10/group) were injected with a lethal dose of intraperitoneal (i.p.) LPS (10 mg/kg b.w.) and D-(+)-galactosamine hydrochloride (500 mg/kg b.w.). Two hours later, mice were injected with a single, 0.1 mg/kg b.w. of i.t. eCS. The mortality of mice was monitored for 8 days.

### Myeloperoxidase (MPO) activity

Mouse lung homogenate was prepared, with which MPO activity was determined by the myeloperoxidase fluorometric detection kit and the manufacturer protocol (Enzo Life Sciences Inc., New York, USA).

### Statistical analysis

One-way analysis of variance (ANOVA) along with Tukey’s post hoc test was used to compare among groups (InStat, Graphpad Software, Inc., CA, USA). *P* values less than 0.05 are considered of statistical significance, for which experiments were performed three times independently.

## Results

### Cytotoxicity of eCS

**Fig. 1:**
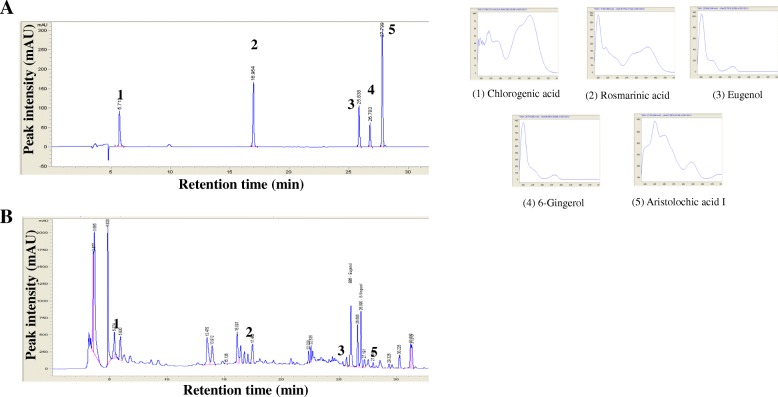
Fingerprinting of eCS. **a** The representative chromatogram used as standard markers is shown: chlorogenic acid (1) of Xanthii Fructus, rosmarinic acid (2) of Menthae Herba, eugenol (3) of Syzygii Flos, 6-gingerol (4) of Zingiberis Rhizoma Crudus, and aristolochic acid I (5) of Asiasari Radix. The key marker compounds detected in the 50% ethanol extract of CS (eCS) are shown in (**b**)

**Fig. 2:**
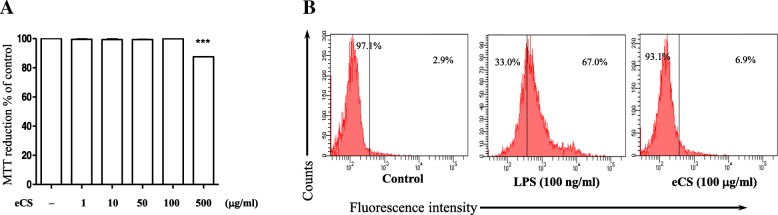
Cytotoxicity of eCS. Cytotoxicity induced by eCS (**a**) was determined by an MTT assay using RAW 264.7 cells. Data represent the mean ± SEM in triplicate. ***P was less than 0.0001, compared to the untreated control. **b** Intracellular ROS generated in RAW 264.7 cells after treated with eCS (100 μg/ml) for 16 h were measured by a flow cytometer. Treatment with LPS (100 ng/ml) for 16 h was included as a positive control for intracellular ROS. The percentiles of ROS-positive cells are shown in the right columns

The powder of the ethanol extract of CS (eCS) was suspended in PBS and filter-sterilized prior to experiment. The fingerprinting of eCS was performed by HPLC to obtain a chemical profile, along with key index chemicals that are suggested by Korean Pharmacopoeia issued by Korea Food and Drug Administration (Fig. 1). We used the profile and the index chemicals of eCS as a reference to ensure the consistency of eCS between batches. Since eCS is a new formula, we determined a possible cytotoxic effect of eCS. RAW 264.7 cells were treated with 1 μg/ml to 500 μg/ml of eCS. At 16 h after treatment, an MTT assay was performed. As shown in Fig. 2a, eCS showed no significant cytotoxicity to RAW 264.7 cells, except at 500 μg/ml of eCS. Given that ROS inflict damage to the host cells, we also examined whether eCS induces the production of intracellular ROS, contributing to cytotoxicity. RAW 264.7 cells were treated with 100 μg/ml of eCS, at which cytotoxicity was not apparent in the MTT assay. At 16 h after treatment, the intracellular ROS were measured by a flow cytometer. As shown in Fig. 2b, while LPS induced the production of intracellular ROS, eCS did not significantly increase intracellular ROS. As a lower dose of eCS was preferable, we used less than 10 μg/ml of eCS for the study.

### eCS did not suppress NF-κB

**Fig. 3:**
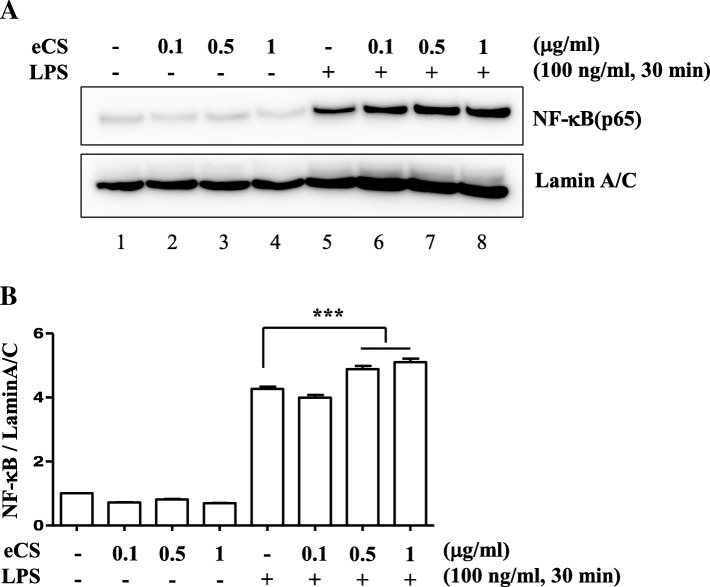
The effect of eCS on NF-κB activity. **a** Pre-treated with eCS for 16 h, RAW 264.7 cells were further treated with TLR4 specific LPS (100 ng/ml) for 30 min. Nuclear proteins were fractionated and analyzed by immunoblotting for p65 RelA, a key subunit of NF-κB. The membrane was stripped and blotted for Lamin A/C as for an internal control for nuclear proteins. Each band on the blots was analyzed by ImageJ, a densitometric analysis program (**b**). The relative levels of nuclear p65 RelA were calculated over Lamin A/C. ***P was less than 0.0001, compared to the LPS-treated control. Data are presented as the mean ± SEM of 3 measurements. At least, two more similar experiments were performed independently and a representative result is shown

Since eCS is composed of the herbs that have anti-inflammatory effects (Table 1), we tested the possibility that eCS exerts an anti-inflammatory function by suppressing NF-κB, a major transcription factor that promotes inflammatory responses [21]. RAW 264.7 cells were treated with three different amounts of eCS, 0.1 μg/ml, 0.5 μg/ml, and 1 μg/ml, for 16 h and subsequently with 0.1 μg/ml of TLR4-specific LPS for 30 min. Nuclear fractions of variously treated cells were prepared (Fig. 3c) and analyzed by immunoblotting for p65 RelA, a subunit of NF-κB [22]. As shown in Fig. 3a, LPS treatment elicited nuclear localization of NF-κB (lane 5), indicative of NF-κB activation, which was however not suppressed by eCS (lanes 6 to 8). The densitometric analysis shows that eCS at high amounts rather slightly activated NF-κB (Fig. 3b). Nevertheless, these results suggest that eCS does not suppress NF-κB activity.

### eCS activated Nrf2, which was associated with a decreased ubiquitination of Nrf2

**Fig. 4:**
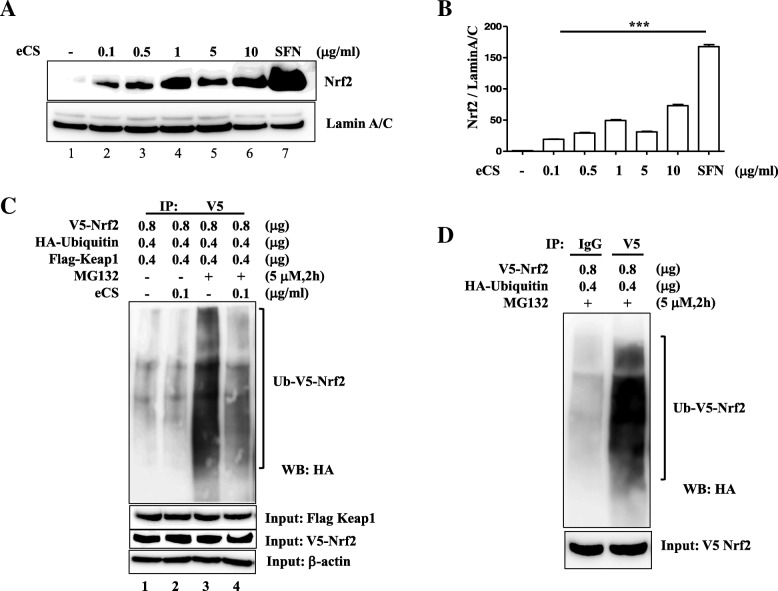
The effect of eCS on Nrf2 activity. **a** Nuclear proteins were fractionated from RAW 264.7 cells treated with various amounts of eCS for 16 h and then analyzed by immunoblotting for Nrf2. **b** The relative amounts of nuclear Nrf2 were calculated over Lamin A/C using ImageJ. ***P was less than 0.0001, compared to the untreated control. Data are presented as the mean ± SEM of 3 measurements, and a representative of 3 independent experiments is shown. **c** HEK 293 cells were transfected with V5-Nrf2, HA-Ub, and Flag-Keap1 and then treated with eCS for 16 h with or without MG132 (5 μM, 2 h). V5-Nrf2 in the total cell lysate was precipitated with the anti-V5 antibody and the precipitant was analyzed with the anti-HA antibody to reveal the ubiquitinated Nrf2. One-tenth of total cell lysate was analyzed for Flag (Keap1), V5 (Nrf2), and β-actin, as inputs. **d** Similarly, HEK 293 cells were transfected with V5-Nrf2 and HA-Ub, along with MG132 (5 μM, 2 h). V5-Nrf2 in the total cell lysate was precipitated with an isotypic IgG or the anti-V5 antibody. The precipitants were analyzed with the anti-HA antibody to reveal the ubiquitinated Nrf2. One-tenth of total cell lysate was analyzed for V5-Nrf2 as in a input

Given that Nrf2 has been known as a critical anti-inflammatory factor, we tested whether eCS activates Nrf2. Similar to the experiments described above, RAW264.7 cells were treated with 0.1 μg/ml to 10 μg/ml of eCS for 16 h, and then nuclear proteins were isolated and analyzed by immunoblotting for nuclear Nrf2, indicative of Nrf2 activation [10]. As shown in Fig. 4a, eCS increased the level of the nuclear Nrf2 (lanes 2 to 6), which occurred as low as 0.1 μg/ml of eCS (lane 2). Compared with sulforaphane, a potent activator of Nrf2 [23] (lane 7), these results suggest that eCS activates Nrf2. Densitometric analyses show that eCS significantly activated Nrf2 (Fig. 4b).

Activation of Nrf2 is inversely related to the level of ubiquitinated Nrf2 [10]. Therefore, we tested whether eCS suppresses the ubiquitination of Nrf2. HEK 293 cells were transfected with plasmids encoding V5-Nrf2, HA-ubiquitin, and Flag-Keap1 for 48 h and then treated with 0.1 μg/ml of eCS for 16 h, with or without MG132 (5 μM), a proteasome inhibitor that will block the degradation of ubiquitinated proteins. An antibody against V5 (Fig. 4c) or an isotypic IgG (Fig. 4d) was added to the total cell lysate. The immune complex was analyzed by immunoblotting for HA (ubiquitin) to reveal the ubiquitinated Nrf2. As shown in Fig. 4c, while Keap1 enhanced the ubiquitination of Nrf2 (lane 3), eCS decreased the level of the ubiquitinated Nrf2 (lane 4). In a similar experiment with isotypic IgG, ubiquitination of Nrf2 was not detectable (Fig. 4d). Combined with Fig. 4a, these results collectively suggest that eCS activating Nrf2 is associated with suppressed ubiquitination of Nrf2.

**Fig. 5:**
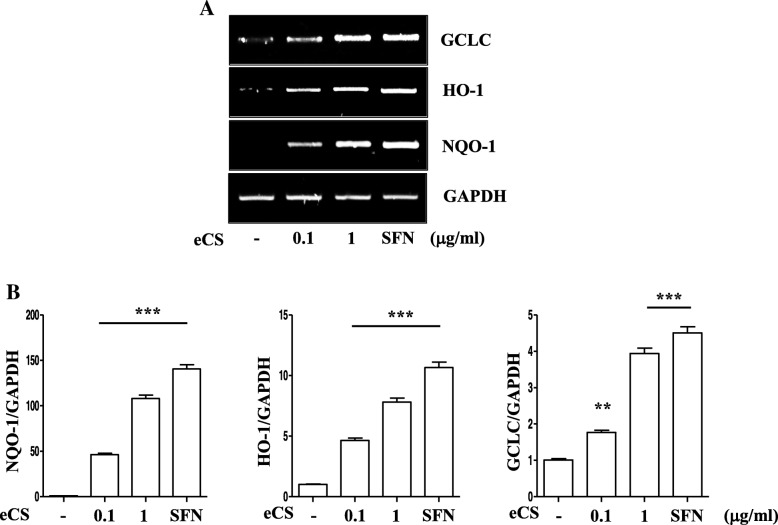
The effect of eCS on the expression of Nrf2-dependent genes. **a** Total RNA was extracted from RAW 264.7 cells treated with eCS for 16 h, and expressions of GCLC, HO-1, and NQO-1 were analyzed by a semi-quantitative RT-PCR. The relative expression of Nrf2-dependent genes was calculated over GAPDH using ImageJ (**b**). **P and ***P were less than 0.001 and 0.0001, respectively, compared to the untreated control. Data are presented as the mean ± SEM of 3 measurements, and a representative result of at least 3 independent experiments is shown

To confirm that eCS activated Nrf2, we examined whether eCS elicits the expression of Nrf2-dependent genes. RAW 264.7 cells were treated with 0.1 μg/ml or 1 μg/ml of eCS for 16 h, from which total RNA was extracted and analyzed by a semi-quantitative RT-PCR for the expression of prototypic Nrf2-dependent genes, such as NQO-1, HO-1, and GCLC [24, 25]. As shown in Fig. 5a, eCS induced the expression of these genes. Densitometric analyses revealed that the expression of those genes was proportionate to increasing amounts of eCS (Fig. 5b). Taken together, these results suggest that eCS suppresses ubiquitination of Nrf2 and activates Nrf2, resulting in the increased expression of Nrf2-dependent genes.

### Intratracheal eCS ameliorated neutrophilic lung inflammation in an ALI mouse model

**Fig. 6:**
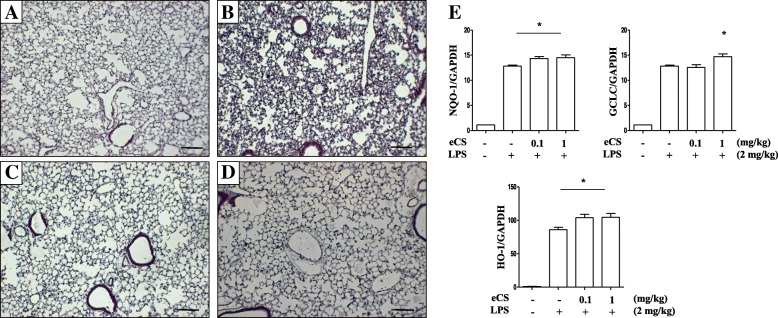
The effect of intratracheal delivery of eCS on lung inflammation and the expression of Nrf2-dependent genes in an LPS-induced ALI mouse model. C57BL/6 mice (*n* = 5/group) received sham (**a**) or a single, 2 mg/kg body weight of i.t. LPS (**b**, **c**, and **d**). At 2 h after LPS treatment, mice received a single, 0.1 mg/kg body weight of i.t. eCS (**c**) or 1 mg/kg body weight of i.t. eCS (**d**). At 24 h after LPS administration, the lungs of mice were harvested and stained with HE for histological examination. Data are representatives of at least five different areas of a lung (bar, 200× magnifications). **e** Total RNA extracted from the harvested lungs (n = 5/group) was analyzed by semi-quantitative RT-PCR to assess expressions of NQO-1, HO-1, and GCLC. The intensity of each PCR band was measured by densitometric analysis (ImageJ) and normalized to GAPDH intensity. * P was less than 0.05, compared with the LPS treated (post-ANOVA comparison with Tukey’s post hoc test)

Given that eCS activated Nrf2 that protects mice from acute lung injury (ALI) [26] and ALI is a representative inflammatory lung disease [27], we set out an ALI mouse model to test whether eCS suppresses neutrophilic lung inflammation, a hallmark of ALI [27]. Since 0.1 μg/ml or 1 μg/ml of eCS activated Nrf2 in RAW 264.7 cells, we tested 0.1 mg/kg and 1 mg/kg body weight of eCS in mice. C57BL/6 mice (*n* = 5/group) received a single i.t. LPS (2 mg/kg body weight) and 2 h later the two different doses of a single i.t. eCS. At 24 h after LPS administration, mice were sacrificed for analyses. Histologic analyses of lung tissue show that, unlike sham-treated controls (Fig. 6a), a single i.t. LPS caused increased cellularity in the airspace of the lung, as indicated by a high number of nuclei, and alveoli thickness with some hyaline changes (Fig. 6b). However, these changes caused by i.t. LPS were relieved after administrating 0.1 mg/kg body weight (Fig. 6c) or 1 mg/kg body weight (Fig. 6d) of eCS, which was comparable to the sham-control (Fig. 6a).

Since eCS activated Nrf2 and induced the expression of Nrf2-dependent genes in RAW 264.7 cells (Fig. 5), we examined whether eCS similarly increases the expression of Nrf2-dependent genes in the lung by a semi-quantitative RT PCR. As shown in Fig. 6e, eCS treatment further increased the expression of NQO-1, HO-1, and GCLC (2nd, 3rd, and 4th columns) with a statistic significance, suggesting that eCS increasing Nrf2-dependent genes are associated with decreased lung inflammation.

**Fig. 7:**
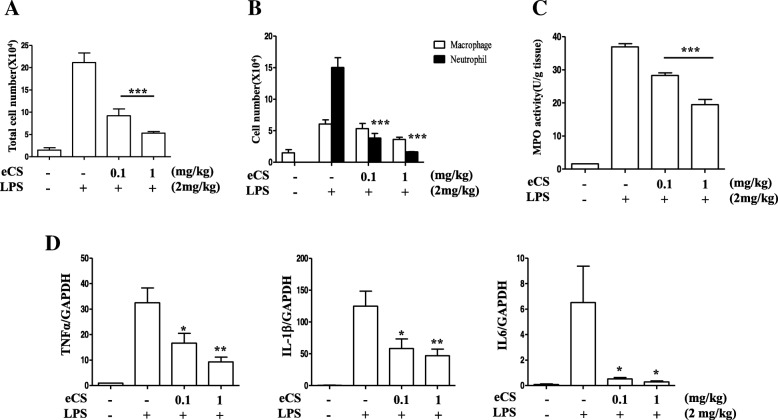
Intratracheal eCS suppresses neutrophil infiltration in mouse lungs. Bronchoalveolar lavage (BAL) was performed with C57BL/6 mice (n = 5/group) treated as in Fig. 5. Total cells (**a**) and macrophages (open columns) and neutrophils (closed columns) (**b**) were counted in the BAL fluid. ***P was less than 0.0001, compared to the mice treated with LPS only. **c** After perfusion, lung lysate was prepared, with which MPO activity was measured. ***P was less than 0.0001, compared to the mice treated with LPS only. Data are presented as the mean ± SEM of 5 mice per group. **d** Total RNA was extracted from the lung, with which expressions of IL-6, IL-1β, and TNF-α were analyzed by a real-time quantitative PCR. *P and **P were less than 0.05 and 0.001, respectively, compared to the mice treated with LPS only

To confirm the suppressive effect of eCS on lung inflammation, we performed broncho-alveolar lavage (BAL) and counted total cells in BAL fluid. As shown in Fig. 7a, while an i.t. LPS increased cell infiltration to the lung (2nd column), eCS significantly suppressed it (3rd and 4th columns). Differential cell counting reveals that the major cell type in the lung after LPS administration was neutrophils (2nd filled column in Fig. 7b), the filtration of which was, however, significantly suppressed by 0.1 mg/kg (3rd filled column) and to a higher degree by 1 mg/kg body weight of eCS (4th filled column). As myeloperoxidase (MPO) is peculiar to neutrophils [6], we tested whether eCS similarly decreases the MPO activity. As shown in Fig. 7c, MPO activity increased by i.t. LPS (2nd column) was similarly decreased by eCS (3rd and 4th columns), suggesting that eCS significantly suppresses neutrophilic lung inflammation. Furthermore, we examined whether eCS down-regulates the expression of representative pro-inflammatory genes, including IL-6, IL-1β, and TNFα. Total RNA was extracted from the lungs of mice (*n* = 5/ group) treated as in Fig. 5 and analyzed by a real-time quantitative PCR. As shown in Fig. 7d, eCS significantly suppressed the expressions of IL-6, IL-1β, and TNFα. Together, our results show that eCS suppressed the neutrophilic lung inflammation in an LPS induced ALI mouse model.

### Intratracheal eCS protected mice from sepsis

**Fig. 8:**
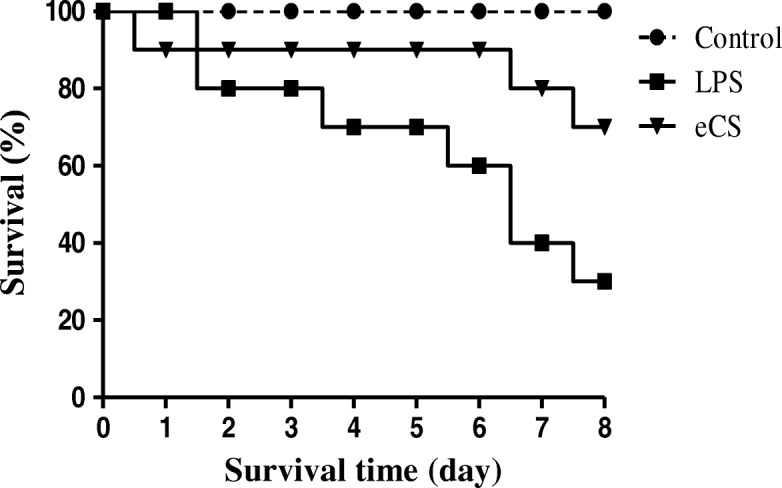
Intratracheal eCS reduces the mortality of mice caused by sepsis. C57BL/6 (*n* = 10/group) received a single i.p. PBS () or of LPS/d-(+)-galactosamine hydrochloride, without () or with a single i.t. eCS (0.1 mg/kg body weight) 2 h later (). Morbidity and mortality were monitored for 8 days. The results are represented by Kaplan–Meier survival curves (log-rank test, * *P* < 0.05)

As sepsis is the major cause of a severe form of acute lung inflammation [5], we tested whether eCS protects mice from succumbing to sepsis (Fig. 8). C57BL/6 mice (*n* = 10/group) received a single i.p. injection of PBS () or of LPS (10 mg/kg body weight) with d-(+)-galactosamine hydrochloride (500 mg/kg body weight) (). At 2 h after injection, mice were administered with either a single i.t. PBS () or i.t. eCS (0.1 mg/kg body weight) (). Mice were closely monitored for mortality for 8 days. As shown in Fig. 7, while control mice showed no mortality (), the mice that received LPS and d-(+)-galactosamine were progressively morbid and 70% of mice were dead by day 8 (). However, when administered with i.t. eCS, the mortality of the mice that received both LPS and d-(+)-galactosamine was 10% until day 6 and increased to 30% by day 8 (). These results show that eCS could protect mice from succumbing to sepsis, suggesting that eCS has a therapeutic potential in suppressing a more severe form of lung inflammation.

## Discussion

Although herbal remedy is the mainstay of Asian traditional medicine including KTM, the traditional herbal remedy is, in general, bulky as for a single dose and relatively slow-acting. This could be due to the way of formulating the remedy: one or two key herbs and other secondary herbs that help complement the therapeutic function of the key herb. Here, we explored the possibility that an herbal remedy composed of major herbs only has a therapeutic efficacy without significant adverse effect. To this end, we formulated an experimental herbal medicine, Chung-Sang (CS), which consisted of five major herbs that have been used to treat inflammatory symptoms and tested whether CS is effective in treating respiratory diseases compounded by inflammation. Our results show that while no significant cytotoxicity was detected, the 50% ethanol extract of CS (eCS) activated Nrf2 by inhibiting the ubiquitination of Nrf2 and induced Nrf2-dependent gene expression. In an ALI mouse model, low amounts of a single i.t. eCS, 0.1 mg/kg or 1 mg/kg body weight, suppressed neutrophilic lung inflammation. Furthermore, 0.1 mg/kg body weight of a single i.t. eCS significantly protected mice from succumbing to sepsis, a cause of a severe form of lung inflammation. Together, our results suggest that eCS effectively suppresses lung inflammation, which was associated, at least in part, with eCS activating Nrf2.

Prior to this study, we prepared two different extracts of CS: the conventional water and 50% ethanol extracts (eCS). Unlike our expectation, the water extract exhibited more cytotoxicity to cells and morbidity in mice (unpublished data), which prompted us to study eCS over the water extract of CS. When formulating CS, we would like to develop a new formula that treats respiratory diseases ridden by inflammation. Therefore, the herbs constituting CS have been known to suppress inflammation and bacterial infection. Since NF-κB is a key factor in promoting lung inflammation [28], we first tested whether eCS suppresses NF-κB activity, contributing to suppression of inflammation. However, we found no evidence that eCS suppressed NF-κB activity in our experimental settings. Despite the result, it should be noted that we cannot exclude the possibility that eCS suppresses NF-κB activity at higher amounts. Since eCS is a concoction of five herbs with anti-inflammatory activity, we presumed that eCS might have a strong anti-inflammatory activity and thus we used only a microgram range of eCS, from 0.1 μg/ml to 1 μg/ml. The possibility that at higher amounts, eCS could suppress NF-κB activity is open and likely.

**Fig. 9:**
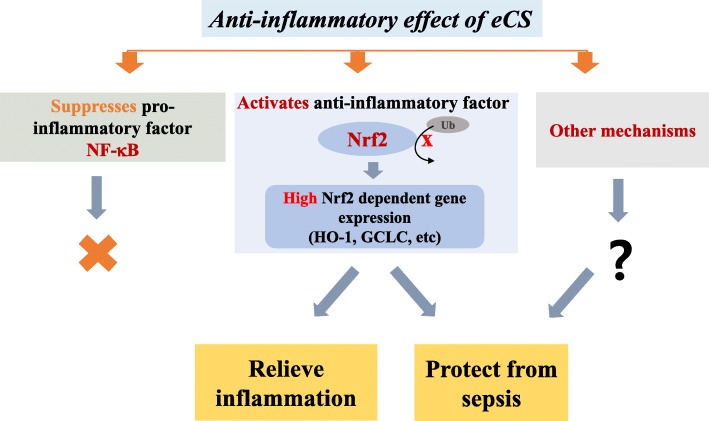
Schematic mechanisms of eCS in suppressing inflammation. The anti-inflammatory activity of eCS could be achieved by suppressing a pro-inflammatory factor, NF-κB, and/or activating an anti-inflammatory factor, Nrf2. In our experimental setting, eCS activated Nrf2, while seemingly not suppressing NF-κB. Alternative pathways are likely and need to be explored. Regardless of mechanisms in detail, eCS effectively suppressed acute and severe lung inflammations in mice

Our results show that micromolar amounts of eCS activated Nrf2, a potent anti-inflammatory factor [29]. In line with this finding, the low amount of eCS robustly induced the expression of NQO-1, GCLC, and HO-1, prototypic Nrf2-dependent genes [30]. Activation of Nrf2 by eCS was corroborated by the results showing that eCS inhibited the ubiquitination of Nrf2 because the degree of the ubiquitination of Nrf2 is inversely correlated with the activation of Nrf2 [31, 32]. It is notable that eCS activating Nrf2 occurred without generating ROS, which is known to activate Nrf2 by inactivating Keap1 [10, 11], suggesting that eCS directly activates Nrf2. Given our result that eCS suppressed the ubiquitination of Nrf2, which is mediated by Keap1, it is conceivable that some chemicals in eCS bind to key cysteine residues at 151, 273, or 288 in Keap1 where major chemical modifications occur [33], resulting in inactivation of Keap1 and thus suppression of the ubiquitination of Nrf2. Given the multitude of chemicals constituting eCS, it is highly likely that eCS activating Nrf2 is a part of mechanisms that bestow an anti-inflammatory function to eCS. In addition, other mechanisms that enable eCS to suppress inflammation are likely feasible (Fig. 9). Nevertheless, it would be interesting to find whether eCS induces chemical modifications at those residues, which would give us an insight on how eCS suppressed the ubiquitination of Nrf2 and thus activated Nrf2.

Although Nrf2 is a key transcription factor that suppresses inflammation, it would be necessary to demonstrate that eCS suppresses inflammation in mice because inflammation is a complex innate immune response that involves various cell types in an organism [8]. Therefore, we tested the anti-inflammatory effect of eCS by using an LPS-induced ALI mouse model. It appears that a single i.t. administration of eCS (0.1 mg/kg body weight) was sufficient to suppress the infiltration of neutrophils to the lung, with the concomitant reduction of the expression of pro-inflammatory genes, such as IL-1β, TNF-α, and IL-6. Suppression of neutrophil infiltration to the lung was further confirmed by MPO assay, which shows that eCS suppressed MPO activity in mouse lungs. Since neutrophilic lung inflammation is a hallmark of ALI and sepsis [34], we further examined whether eCS is also effective in protecting mice from sepsis. We found that a single administration of 0.1 mg/kg body weight of i.t. eCS could reduce the mortality from 70 to 30% by day 8 after the onset of sepsis. Given that a low amount of eCS significantly suppressed acute lung inflammation in ALI and mortality from sepsis, it is likely that eCS can be developed as a potent anti-inflammatory herbal remedy.

The high potency of eCS in suppressing inflammation observed in this study could be attributable to the route of eCS delivery. Unlike the conventional oral taking of herbal remedy, in this study, eCS was delivered in aerosol directly to the lung. While oral administration makes a drug exert its effect in a systematic fashion and thus it takes time for a full pharmaceutical function, intratracheal administration of eCS that targets the lung might allow eCS to work rather quickly. In fact, this way of delivery contributes to the increased efficacy of a drug [35]. Thus, in addition to the potency of eCS, the efficacy of eCS in suppressing inflammation could be further increased, at least in part, by the direct delivery of eCS to the lung.

One of the major roles of complementary herbs is to balance out any side effect inflicted by the major herb in an herbal remedy. To our knowledge, formulating an herbal remedy without containing complementary herbs has been less explored and the experimental basis of using complementary herbs is rather obscure. We assumed that if a major herb does not show an adverse effect, then complementary herbs that would decrease the side effect of the major herb might not be necessary for the formula. In addition, omitting complementary herbs could provide a leeway to make up an herbal formula. For instance, it will reduce the overall size of a single dose, which is less bulky than conventional formulas. In the place of complementary herbs, other major herbs with similar therapeutic effects could be added, enhancing the pharmaceutical effectiveness of the major herbs. In this study, we formulated eCS accordingly and observed that a low dose of eCS was effective in decreasing neutrophilic lung inflammation and protecting from sepsis that causes a serious, more severe form of lung inflammation. During the experiment, no morbidity caused by eCS alone was detectable in mice. Therefore, our results highlight the feasibility of formulating herbal remedy that is composed of major herbs without complementary herbs.

## Conclusions

A small quantity of eCS suppressed lung inflammation in an ALI mouse model and protected mice from sepsis, which was attributable, at least in part, to eCS activating Nrf2, but not significantly to suppressing NF-κB (Fig. 9). Our results support the possibility that a formula composed of the major herbs with a similar therapeutic effect can be developed as an alternative to conventional herbal remedies.

## Abbreviations

HPLC: High-performance liquid chromatography
MPO: Myeloperoxidase
MTT: (3-(4,5-Dimethylthiazol-2-yl)-2,5-Diphenyltetrazolium Bromide)
NF-κB: nuclear factor kappa-light-chain-enhancer of activated B cells
Nrf2: Nuclear factor (erythroid-derived 2)-like 2
qPCR: Quantitative polymerase chain reaction
